# Catheter, MRI and CT Imaging in Newborns with Pulmonary Atresia with Ventricular Septal Defect and Aortopulmonary Collaterals: Quantifying the Risks of Radiation Dose and Anaesthetic Time

**DOI:** 10.1007/s00246-018-1895-7

**Published:** 2018-05-09

**Authors:** David F. A. Lloyd, Sebastian Goreczny, Conal Austin, Tarique Hussain, Shakeel A. Qureshi, Eric Rosenthal, Thomas Krasemann

**Affiliations:** 1 Department of Congenital Heart Disease, Evelina Children’s Hospital, London, UK; 2 Division of Pediatric Cardiology, Department of Pediatrics, Erasmus MC Rotterdam, Sophia Kinderziekenhuis, Wytemaweg 80, 3015CN Rotterdam, The Netherlands

**Keywords:** Pulmonary atresia, Aortopulmonary collaterals, Imaging modalities, Radiation, Anesthetic time

## Abstract

A comprehensive understanding of the native pulmonary blood supply is crucial in newborns with pulmonary atresia with ventricular septal defect and aortopulmonary collaterals (PA/VSD/MAPCA). We sought to describe the accuracy in terms of identifying native pulmonary arteries, radiation dose and anaesthetic time associated with multi-modality imaging in these patients, prior to their first therapeutic intervention. Furthermore, we wanted to evaluate the cumulative radiations dose and anaesthetic time over the study period. Patients with PA/VSD/MAPCA diagnosed at < 100 days between 2004 and 2014 were identified. Cumulative radiation dose and anaesthetic times were calculated, with imaging results compared with intraoperative findings. We then calculated the cumulative risks to date for all surviving children. Of 19 eligible patients, 2 had echocardiography only prior to first intervention. The remaining 17 patients underwent 13 MRIs, 4 CT scans and 13 cardiac catheterization procedures. The mean radiation dose was 169 mGy cm^2^ (47–461 mGy cm^2^), and mean anaesthetic time was 111 min (33–185 min). 3 children had MRI only with no radiation exposure, and one child had CT only with no anaesthetic. Early cross-sectional imaging allowed for delayed catheterisation, but without significantly reducing radiation burden or anaesthetic time. The maximum cumulative radiation dose was 8022 mGy cm^2^ in a 6-year-old patient and 1263 min of anaesthetic at 5 years. There is the potential to generate very high radiation doses and anaesthetic times from diagnostic imaging alone in these patients. As survival continues to improve in many congenital heart defects, the important risks of serial diagnostic imaging must be considered when planning long-term management.

## Introduction

Pulmonary atresia with ventricular septal defect represents a spectrum of congenital heart disease with significant anatomical heterogeneity. In patients where the pulmonary blood supply is provided by aortopulmonary collaterals(PA/VSD/MAPCA), accurate imaging is critical to long term planning and prognosis; in particular, the presence of absence of native pulmonary arteries [1, 2]. In view of this, multiple imaging modalities may be employed in the same patient even before any intervention is performed, all of which can carry important risks (Table 1) [3–9]. General anaesthesia, for example, almost universally required under 6 months of age, carries significant risks in patients with single ventricle physiology (such as pulmonary atresia) in this age group [10–13]. The use of ionising radiation associated with CT and cardiac catheterisation also carries important long-term risks, with younger children 3 to 4 times more likely than adults to develop malignancies following radiation exposure [3, 14–16]. Cardiac catheterisation alone accounts for by far the largest proportion of radiation exposure in children with congenital heart disease [3, 17, 18].

**Table 1:** Imaging modalities in patients with pulmonary atresia

Modality	Advantages	Disadvantages/risks
Echocardiography	Bedside test Non-invasive	Poor visualisation of most extrapericardial structures
Cardiac catheterisation [4, 5, 8]	Can determine dual supply of lung segments Direct pressure measurements Accurate in identifying native pulmonary arteries	Invasive Risk of vascular injury, stroke, death General anaesthetic required Radiation risk
CT Angiography [4, 6, 7]	Fast acquisition Accurate for native pulmonary arteries, shunts and vessel sizes Can image extracardiac structures	General anaesthetic likely to be required Radiation risk
MRI Angiography [6, 8, 9]	Relatively accurate for pulmonary arteries and larger collaterals Can calculate flow rates Can image extracardiac structures No radiation	General anaesthetic likely to be required Less accurate than CT for sub-millimetre vessels Slow acquisition time Possible gadolinium deposition in the brain

The aim of this study was to quantify the radiation exposure and anaesthetic time associated solely with diagnostic imaging to identify native pulmonary arteries, if present, in newborns and infants with PA/VSD/MAPCA, prior to their first therapeutic intervention. We then calculated the cumulative risks to date for all surviving children.

## Materials and Methods

The institutional database of the Department of Congenital Heart Disease (Heartsuite, Systeria, Glasgow, United Kingdom), at the Evelina London Children’s Hospital in London, United Kingdom, was interrogated to find all patients diagnosed with pulmonary atresia, ventricular septal defect and aortopulmonary collaterals under 100 days of age, between 2004 and 2014. The cumulative radiation doses and anaesthetic time of patients during the study period was calculated. Radiation doses are given in dose area product units (cGy cm^2^) [16], which are independent of the location of measurement and regarded as being suitable for describing radiation exposure in children [19].

In keeping with unit policy, decision making for these complex patients was not protocolised over this time period, and imaging strategies were determined on a case-by-case basis; hence, not every patient underwent all imaging modalities. We also evaluated the cumulative radiation dose and anaesthetic time for these patients over the whole study period.

## Results

**Fig. 1:**
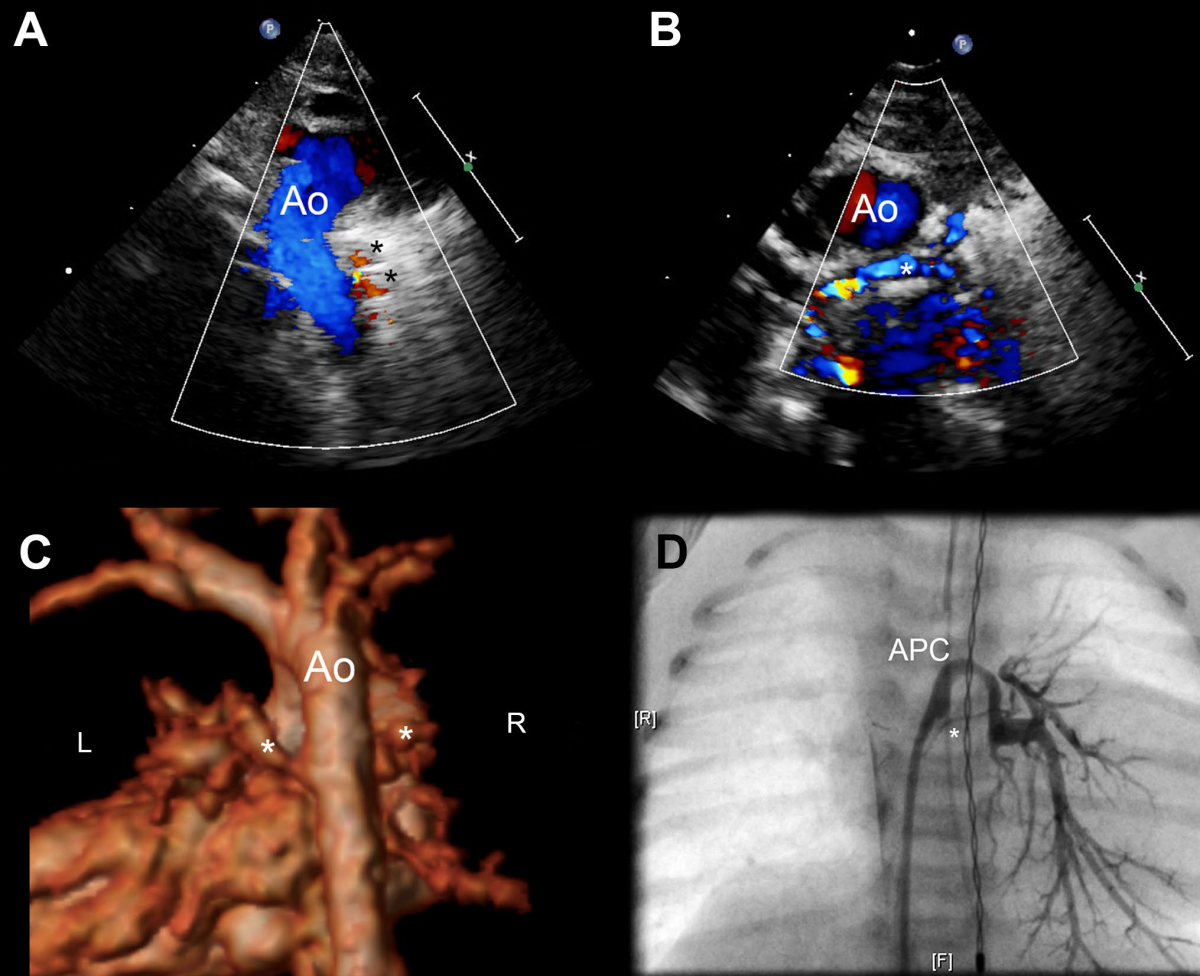
**a** Echocardiography immediately after birth in a patient antenatally diagnosed with pulmonary atresia and ventricular septal defect showing a right sided aortic arch and collateral vessels (asterisked) arising from the descending aorta. *Ao* aorta. **b** Parasternal short axis view from the same study showing suspected confluent, but severely hypoplastic pulmonary arteries (asterisked). *Ao* aorta. **c** 3D reconstructed MRI on day 1 of life in the same patient, clearly demonstrating large collaterals to the left and right lung from the descending aorta. The possibility of a small native left pulmonary artery was raised but the study was not conclusive. CT imaging was subsequently performed at 7 days—no native pulmonary arteries were identified. **d** Still from aortic injection during cardiac catheterisation on day 13 of life. Tiny confluent branch pulmonary arteries were identified (asterisked), in addition to the major collaterals previous described

**Fig. 2:**
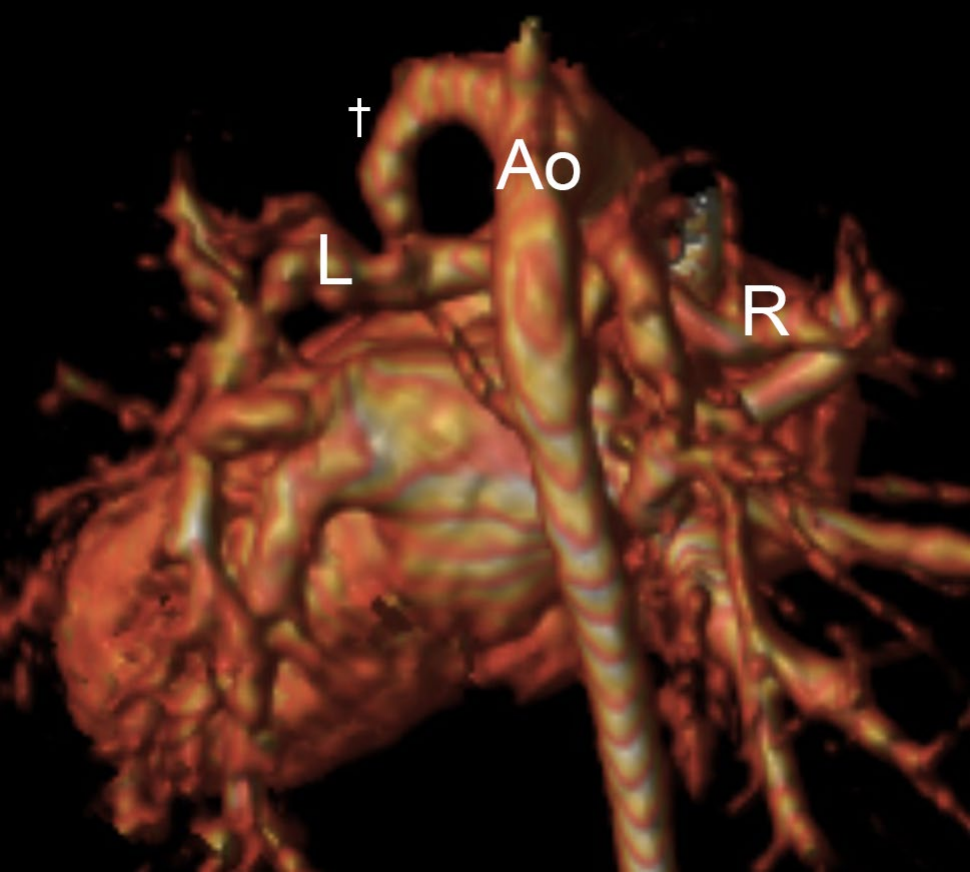
3D reconstructed MRI at 9 months of age, following the insertion of a left-sided Blalock-Taussig shunt (†) at 34 days of age. The right (R) and left (L) pulmonary arteries are now clearly visible. Prior to intervention, the patient had received a total of 120 min of general anaesthetic time and a radiation dose of 191 mGy m^2^. *Ao* aorta

19 patients diagnosed with PA/VSD/MAPCA under the age of 100 days were identified. The first investigation was transthoracic echocardiography in all cases, of which 17 patients went on to have further imaging. In total, there were 13 MRI scans, 13 cardiac catheterisations and 4 CT scans performed in this group before therapeutic intervention. All were within the first 100 days of life, and aside from one patient with 2 MRI scans, no patient had the same investigation more than once. A full summary of the imaging strategy used in each patient is depicted in Table 2, including the accuracy of the imaging modalities in identifying the presence of native pulmonary arteries. Example imaging from a single patient is shown Figs. 1 and 2.

**Table 2:** Imaging strategy, additional findings and cumulative radiation dose and anaesthetic time prior to first intervention

	Age (d)	PAs on echo?	Imaging 1	Age (d)	PAs?	Imaging 2	Age (d)	PAs?	Other findings	Imaging 3	Age (d)	PAs?	Other findings	PAs at Surgery?	GA (mins)	Rad (mGy cm^2^)	Surgery
1	0	Yes	–	–	–	–	–	–	–	–	–	–	–	Yes	0	0	Shunt to PAs
2	0	Yes	Cath	6	Yes	–	–	–	–	–	–	–	–	Yes	181	78	Shunt to PAs
3	1	No	MRI	5	No	MRI	79	No	+ 1 APC	Cath	86	No	None	No	90	141	Shunt to unif.
4	0	Yes	MRI	4	Yes	–	–	–	–	–	–	–	–	Yes	92	0	Shunt to PAs
5	0	Yes	MRI	5	Yes	–	–	–	–	–	–	–	–	Yes	33	0	Shunt to PAs
6	0	No	Cath	1	Yes	–	–	–	–	–	–	–	–	Yes	40	62	Shunt to PAs
7	0	Yes	MRI	2	Yes	Cath	16	Yes	None	–	–	–	–	Yes	114	231	Shunt to PAs
8	0	Yes	MRI	19	Yes	Cath	34	Yes	None	–	–	–	–	Yes	185	172	Shunt to PAs
9	0	Yes	Cath	2	Yes	–	–	–	–	–	–	–	–	Yes	108	122	Shunt to PAs
10	0	Yes	MRI	11	No	Cath	34	Yes	None	–	–	–	–	Yes	91	58	Shunt to PAs
11	94	Yes	MRI	96	No	–	–	–	–	–	–	–	–	No	52	0	Shunt to unif.
12	1	No	MRI	6	No	Cath	8	Yes	+ 1 APC	–	–	–	–	Yes	107	158	Shunt to PAs
13	73	Yes	MRI	76	Yes	Cath	80	Yes	+ 1 APC	–	–	–	–	Yes	118	47	Shunt to PAs
14	11	Yes	MRI	18	No	Cath	60	No	None	CT	85	Yes	None	No	157	461	Shunt to unif.
15	10	Yes	MRI	11	No	Cath	17	Yes	+ 1 APC	–	–	–	–	Yes	174	338	Stent to PDA
16	0	Yes	CT	1	Yes	–	–	–	–	–	–	–	–	Yes	0	66	Shunt to PAs
17	0	Yes	MRI	1	No	CT	7	No	+ 2 APCs	Cath	13	Yes	− 1 APC	Yes	130	191	Shunt to PAs
18	23	No	CT	24	No	Cath	25	Yes	PAs	–	–	–	–	Yes	108	238	Conduit to unif.
19	0	Yes	–	–	–	–	–	–	–	–	–	–	–	Yes	0	0	Shunt to PAs

*PAs* pulmonary arteries, *GA* general anaesthetic, *Rad* radiation dose, *Cath* catheterisation, *MRI* magnetic resonance imaging, *APC* aortopulmonary collaterals, *Unif* unifocalised collaterals, *CT* computed tomography, *PDA* patent arterial duct

The mean cumulative anaesthetic time for all forms of imaging was 111 min (median 108 min, range 33–185 min). One child who underwent CT only after echocardiographic evaluation did not have general anaesthetic. The mean radiation dose for the 13 patients undergoing diagnostic cardiac catheterisation was 119 mGy cm^2^ (median 122 mGy cm^2^, range 47–231 mGy cm^2^). In the four patients who underwent CT, the mean radiation dose was 92 mGy cm^2^ (median 90 mGy cm^2^, range 66–123 mGy cm^2^). The mean total radiation dose for the three patients undergoing both catheterisation and CT was 297 mGy cm^2^ (median 238 mGy cm^2^, range 191–461 mGy cm^2^). A total five patients (two with echo only and three echo and MRI only) had no radiation exposure prior to therapeutic intervention.

The average age of patients undergoing primary cardiac catheterisation was 3 days (median 2 days, range 1–6 days, *n* = 3). For patients undergoing primary CT or MRI, the mean age of any subsequent catheterisation (*n* = 10) was 35 days (median 21 days, range 8–86 days; *p* = 0.09). Having an MRI or CT prior to catheterisation did not significantly reduce the radiation dose or anaesthetic time of the cardiac catheterisation in our series.

Over the 10-year period of our study, many surviving patients underwent further imaging to assess the pulmonary vasculature. This comprised of non-invasive cross-sectional imaging as well as diagnostic and/or interventional cardiac catheterisation, most frequently to address circumferential stenosis in the reconstructed pulmonary arteries. Table 3 shows the cumulative number of investigations performed to date, including the total number of X-ray radiological studies, with total radiation dose and anaesthetic times for each patient.

**Table 3:** Lifetime imaging, cumulative radiation dose and anaesthetic time

N	Age at last follow up	*N* Cath	Cath Rad (mGy cm^2^)	Cath GA (mins)	*N* CT	CT Rad (mGy cm^2^)	*N* MRI	MRI GA (mins)	*N* CXR	*N* OXR	Total Rad^a^ (mGy cm^2^)	Total GA (mins)
1	11 years	0	0	0	0	0	2	172	18	34	0	172
2	8 years	3	2843	409	–	–	2	186	34	–	2843	595
3	d. 96 days	1	141	63	–	–	2	57	6	–	141	120
4	5 years	5	4327	811	1	34	4	452	30	6	4361	1263
5	d. 17 months	2	1156	170	–	–	2	96	26	1	1156	266
6	3 years	2	185	125	–	–	2	131	25	1	185	256
7	d. 32 days	1	231	100	–	–	1	14	16	2	231	114
8	2 years	1	172	98	–	–	2	147	15	–	172	245
9	6 years	5	8022	786	–	–	3	358	39	1	8022	1144
10	5 years	3	1357	245	–	–	3	199	45	12	1357	444
11	5 years	2	104	155	1	133	1	52	57	14	237	207
12	d. 18 months	2	1409	164	–	–	2	114	15	1	1409	278
13	4 years	1	47	56	–	–	2	134	23	–	47	190
14	d. 2 years	1	338	108	1	123	2	145	42	4	461	253
15	d. 30 days	1	804	110	–	–	1	64	12	–	804	174
16	16 months	2	349	237	2	404	1	28	26	3	753	265
17	9 months	1	84	119	1	107	1	11	20	7	191	130
18	10 months	1	165	108	2	303	–	–	42	4	468	108
19	d. 31 days	0	0	0	0	0	0	0	0	19	0	0

*N* number, *Cath* catheterisation, *Rad* radiation dose, *CT* computed tomography, *MRI* magnetic resonance imaging, *GA* general anaesthetic, *CXR* chest X-ray, *OXR* other X-ray, *d*. died

^a^Not including radiation from chest and other X-rays

## Discussion

We have attempted to quantify the cumulative anaesthetic time and radiation exposure resulting from serial diagnostic investigations in patients with PA/VSD/MAPCA. The median radiation dose in our series prior to intervention was 122 mGy cm^2^, with one patient undergoing 157 min of general anaesthetic time and a total radiation dose of 461 mGy cm^2^ within the first 3 months of life, prior to any therapeutic intervention being performed. By way of comparison, the median radiation dose for 312 interventional catheter procedures in our institution—across all age groups—was 176 mGy cm^2^ from 2005 to 2009 [16]. We rarely perform pure diagnostic catheterisation in our institution, and hence could not compare to diagnostic catheterisations for other reasons.

As anticipated, despite carrying the highest risks, cardiac catheterisation appeared to be the “gold standard” investigation, correctly identifying the presence or absence of native pulmonary arteries in all patients. It was also most frequently the final investigation before committing to intervention. MRI and CT showed poorer sensitivity to identify native pulmonary arteries in our patients, and whilst the numbers were too small to allow for a comprehensive comparison, falling acquisition times and the use of CT and MRI imaging without anaesthesia continue to increase their attractiveness in a clinical setting [6, 20, 21]. CT in particular has shown promising results for infants with aortopulmonary collaterals [22], with the potential for a reduced radiation burden in modern systems [23]. The potential advantage of cross-sectional imaging providing an initial “roadmap” for subsequent catheterisation was, however, not clearly demonstrated in our series: the mean radiation dose when catheterisation was performed without prior CT/MRI was 87 mGy cm^2^ (median 78 mGy cm^2^, range 62–122 mGy cm^2^, *n* = 3), and 152 mGy cm^2^ (median 150 mGy cm^2^, 47–338 mGy cm^2^, *n* = 10) when cross-sectional imaging was available; the mean anaesthetic time was 110 min (median 108 min, range 40–181 min) versus 86 min (median 99 min, range 40–119 min), respectively (*p* = 0.38). In both MRI and CT settings, correct imaging of vessels is flow dependent, and in cardiac catheterisation usually injections of contrast is done with “power injections” per pump or by hand, hence providing adequate flow locally [24].

Repeated diagnostic and interventional procedures in patients with PA/VSD/MAPCA, in particular cardiac catheterisation, can lead to extremely high cumulative radiation doses in later childhood. Children are more susceptible than adults to the effects of ionising radiation and, as survival continues to improve, these patients will have a longer life-span over which that risk is expressed [16, 25]. One patient of our series has been exposed to a cumulative radiation dose of 8022 mGy/cm^2^ at the age of 6 years, from five catheterisation procedures. One could argue that such radiation dose moves the future malignancy risk from stochastic towards probable, even when taking into account anticipated life expectancy [25].

## Limitations

This is a single centre, retrospective, descriptive study. During the study period, no protocol regarding imaging to identify native pulmonary arteries existed in our institution, and different imaging modalities were applied on a case-to-case basis.

## Conclusion

Whilst optimisation of the pulmonary circulation is crucial in patients with PA/VSD/MAPCA, there is the potential to generate very high radiation doses and anaesthetic times from diagnostic imaging alone. As survival continues to improve in patients with a range of complex congenital heart defects, the important risks of serial diagnostic imaging must be considered alongside long-term interventional strategies.
